# A stochastic differential equation analysis of cerebrospinal fluid dynamics

**DOI:** 10.1186/2045-8118-8-9

**Published:** 2011-01-18

**Authors:** Kalyan Raman

**Affiliations:** 1 Medill IMC Department, Northwestern University, 1870 Campus Drive, Third Floor, Evanston, IL 60208, USA

## Abstract

**Background:**

Clinical measurements of intracranial pressure (ICP) over time show fluctuations around the deterministic time path predicted by a classic mathematical model in hydrocephalus research. Thus an important issue in mathematical research on hydrocephalus remains unaddressed--modeling the effect of noise on CSF dynamics. Our objective is to mathematically model the noise in the data.

**Methods:**

The classic model relating the temporal evolution of ICP in pressure-volume studies to infusions is a nonlinear differential equation based on natural physical analogies between CSF dynamics and an electrical circuit. Brownian motion was incorporated into the differential equation describing CSF dynamics to obtain a nonlinear stochastic differential equation (SDE) that accommodates the fluctuations in ICP.

**Results:**

The SDE is explicitly solved and the dynamic probabilities of exceeding critical levels of ICP under different clinical conditions are computed. A key finding is that the probabilities display strong threshold effects with respect to noise. Above the noise threshold, the probabilities are significantly influenced by the resistance to CSF outflow and the intensity of the noise.

**Conclusions:**

Fluctuations in the CSF formation rate increase fluctuations in the ICP and they should be minimized to lower the patient's risk. The nonlinear SDE provides a scientific methodology for dynamic risk management of patients. The dynamic output of the SDE matches the noisy ICP data generated by the actual intracranial dynamics of patients better than the classic model used in prior research.

## Background

Intracranial dynamics play a central role in healthy brain function because disturbances in the internal fluid environment of the skull can lead to multiple complications such as, among other things, hydrocephalus [1]. Intracranial dynamics, driven by the circulation of CSF, are important because CSF protects the brain from injury, contains nutrients enabling normal functioning of the brain and, transports waste products away from the surrounding tissues. Much more is involved in hydrocephalus than a simple disorder of CSF circulation [2]; it is considered a complex spectrum of diseases, primarily defined by perturbation of the cranial contents--operationalized as CSF volume--and the intracranial pressure [3]. Given the complex nature of hydrocephalus, we define hydrocephalus as a disease associated with disturbances in the CSF dynamics, as in [1].

Experimental evidence compellingly validates that, over a large range of pressures, brain compliance is not constant [4]. Marmarou [5] postulated a hyperbolic compliance function that decreases as the pressure increases, which coupled with other assumptions to be described below, led to a nonlinear ordinary differential equation for the variation of ICP over time. The Marmarou model [5] is fundamental in mathematical pressure-volume models of CSF dynamics. While the Marmarou model has deservedly remained the mainstay of quantitative modeling of the dynamics of CSF flow, its deterministic nature prevents taking full advantage of the information in real ICP measurements, because deterministic models average over all possible fluctuations of real data. The ICP waveform contains additional information that is ignored by the time-averaged ICP mean value [6]. We draw upon the fundamental principles of modeling cerebrospinal fluid dynamics explicated in [2]. Our starting point is Marmorou's model [5,7] of pressure-volume compensation, which was subsequently modified in [8] and [9]. Central to the development of the Marmarou model is a conservation law. Conservation laws are ubiquitous in physics [10]. The Marmarou model represents CSF flow dynamics through a conservation equation relating the production of CSF to its storage and reabsorption [2].

(1) Production of CSF=storage of CSF+reabsorption of CSF

Next, reabsorption is proportional to the differential between CSF pressure (p) and pressure in the sagittal sinuses (p_ss_):

(2) $$\text{reabsorption}=\frac{\text{p}-{\text{p}}_{\text{ss}}}{\text{R}}$$

p_ss _is considered a constant parameter, determined by central venous pressure. The coefficient R is the resistance to CSF reabsorbtion or outflow, measured in units of mmHg mL^-1 ^min. Storage of CSF is proportional to the cerebrospinal compliance C, measured in units of mL mmHg ^-1^.

(3) $$\text{storage}=\text{C}\frac{\text{dp}}{\text{dt}}$$

The compliance of the cerebrospinal space is inversely proportional to the differential of CSF pressure p and the reference pressure p_0 _[8,11], and is considered the most important law of cerebrospinal dynamic compensation [2]:

(4) $$\text{C}=\frac{1}{\text{E}(\text{p}-{\text{p}}_{0})}$$

**Figure 1:**
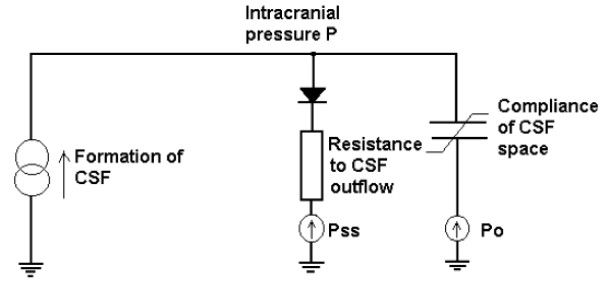
**Electrical circuit analogy for CSF flow dynamics**. The current source reflects formation of CSF; resistor with diode--unilateral absorption of CSF into the sagittal sinuses; capacitor and voltage source--nonlinear compliance of CSF space. P_ss _is the pressure in the sagittal sinuses. P_0 _is the reference pressure. Figure reproduced from [2] with permission.

The coefficient E is called the cerebral elasticity (or elastance coefficient) and has the units mL^-1 ^[12]. Next, by exploiting an analogy between an electrical model of CSF compensation, as described in [5], and adapted in [2], the deterministic description of the dynamics of CSF flow are given by:

(5) $$\frac{1}{\text{E}(\text{p}-{\text{p}}_{0})}\frac{\text{dp}}{\text{dt}}+\frac{\text{p}-{\text{p}}_{\text{b}}}{\text{R}}=\text{I}(\text{t})$$

where I(t) is the rate of external volume addition and p_b _is a baseline pressure. The circuit diagram, reproduced from [2], is shown in Figure 1. An electrical circuit analogy is also used in [13] and [14] to study the dynamics of ICP in the ventricular compartments. The reference pressure parameter p_0 _is sometimes taken to be zero, as for example, in [5] because, as noted in [2], the significance of p_0 _is unclear. Consequently, we assume p_0 _= 0, which results in the equation:

(6) $$\frac{1}{\text{Ep}}\frac{\text{dp}}{\text{dt}}+\frac{\text{p}-{\text{p}}_{\text{b}}}{\text{R}}=\text{I}(\text{t})$$

### The importance of modeling noise in CSF dynamics

**Figure 2:**
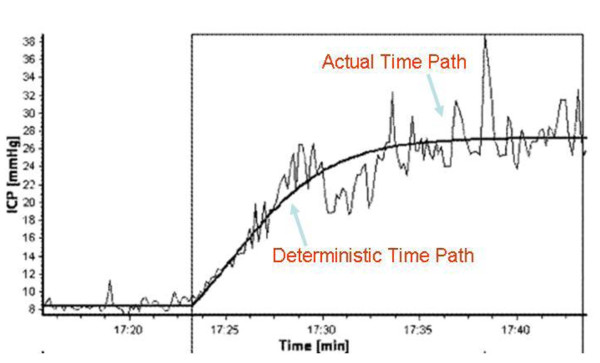
**Comparison of actual path of ICP with the path predicted by the deterministic Marmarou model**. Reproduced from [1] with permission. The smooth curve without any ups and downs is the temporal path of the ICP as predicted by the deterministic Marmarou model. The fluctuations around that deterministic path are due to noise arising from the various sources discussed in the paper.

Broadly construed, noise arises from variations in factors that influence the observed outcome--which is the ICP in this paper-- but that have been omitted from the mathematical model, and from factors affecting the observed outcome that are beyond the experimenter's control. Noise causes deviations of the predicted ICP from the actual ICP level. Factors uncontrolled by the experimenter include thermal fluctuations, body movement and breathing. Because a mathematical model is an abstraction of reality, it is based on simplifying assumptions, as listed in [3]. The Marmarou model abstracts the CSF system as an electrical circuit consisting of a nonlinear capacitor (storage mechanism), resistor (area of CSF absorption), and so on [15]. There remains substantial uncertainty regarding the average rate of CSF production [16]. Realistic estimates of the mechanical properties of the living human brain are hard to discover [15]. The compliance is not an appropriate indicator of the brain's elastic properties [14]. Shunts, used in the treatment of hydrocephalus, can be dramatically improved by more accurate modeling of the CSF dynamics. Shunts providing continuous CSF drainage are the ideal [17], and nonlinear control theory can be used to design an automatic controller for a shunt that provides continuous drainage. But in order to design a stable controller to facilitate a shunt with continuous drainage, we need a model of CSF drainage that either incorporates factors omitted in extant models, or that accounts for the noise caused by the omission. Our objective is to incorporate noise into the dynamics of CSF flow. The effect of noise on the ICP waveform is discernible in Figure 2, which shows the fluctuations of the ICP around the deterministic path predicted by the deterministic Marmarou model.

## Methods

### Generalizing the CSF dynamics to incorporate noisy flow

Visual examination of the time-series of ICP recordings shows that the fluctuations are smooth (unlike electrons in a wire which generate shot noise, characterized by jumps [18]), and therefore continuous state space Markov processes are appropriate to capture the noisy dynamics of CSF flow. A large class of Markov processes can be represented by SDEs, and here a methodological choice must be made--noisy dynamic processes can be represented by stochastic differential equations of the Ito type or the Stratonovich type which correspond to two different ways of introducing noise into a dynamic system. A central difference between the two is that the Stratonovich SDE uses the usual deterministic calculus whereas the Ito SDE requires a completely new stochastic calculus. Extensive conceptual, empirical and philosophical discussions of this issue exist in the literature on mathematical models of electrical, biological and physical phenomena [19-21]. The overwhelming majority of these discussions conclude that Ito processes, generated by stochastic differential equations of the Ito type, are superior to Stratonovich processes, generated by stochastic differential equations of the Stratonovich type [22,23]. Ito [24] extended standard deterministic calculus to a "stochastic calculus" applicable to functions of a wide class of continuous-time random processes, known as Ito processes. Given the SDE for the process under consideration, a result called Ito's Lemma yields the SDE driving the dynamics of a general transformation of the original process [24]. This utilitarian result allows deducing the stochastic properties of considerably complex models driven by Ito processes [23]. An essential property of Ito processes is that nonlinear functions of Ito processes remain Ito processes--a property called closure under nonlinear transformations, indispensable for practical reasons. From an empirical standpoint, a compelling advantage of Ito processes is that they often yield very precise statistical specifications for estimation [23]. An attractive property of Ito processes--on theoretical, mathematical, practical and computational grounds--is that they are Markov processes. Finally, the Ito calculus has been extended to embrace general martingale processes [25]--a development that permits joint consideration of both smooth noise and noise that occurs in jumps. Thus our modeling framework can accommodate neurological phenomena requiring noise that encompasses *both *smooth and jumpy variations in the state of the system, such as the firing of neurons [26].

### Modeling the CSF dynamics as an Ito process to incorporate noisy flow

Given all these considerations, we modeled the fluctuations in CSF dynamics through an Ito stochastic differential equation. First we introduced noise into equation (6) through a white noise process ε(t) with intensity parameter σ, which by definition satisfies the following properties: E[ε(t)] = 0, and E[ε(t) ε(s)] = 0, whenever t ≠ s. The notation E[.] denotes the expectation operator, which, when applied to a random quantity such as ε(t), signifies the value of ε(t) on average. Thus E[ε(t)] = 0 signifies that the average value or mean of the random error at time "t" is zero, and this is a standard assumption in the literature on modeling noisy phenomena. The term E[ε(t) ε(s)] is the expectation operator applied to the product of random errors at two different times 's' and 't;' technically it denotes the covariance between the errors at two different times. In this case, because of the zero-mean assumption, it also denotes the correlation between ε(t) and ε(s); and so the property E[ε(t) ε(s)] = 0 means that the errors at two different times are *uncorrelated*, which substantively means that an error at one point in time does not influence the error at another point in time. This too is a standard assumption in the dynamic modeling literature.

(7) $$\frac{1}{\text{Ep}}\frac{\text{dp}}{\text{dt}}+\frac{\text{p}-{\text{p}}_{\text{b}}}{\text{R}}=\text{I}(\text{t})+\sigma \varepsilon (\text{t})$$

Next we exploited the fundamental relationship between a white noise process ε(t) and a Brownian motion process W(t): $\text{W}(\text{t})=\int_{\text{s}=0}^{\text{s}=\text{t}}\varepsilon (\text{s})\text{ds}\text{a}$, which, when written in differential notation, yields dW = ε(t) dt. Therefore,

(8) $$\frac{1}{\text{Ep}}\frac{\text{dp}}{\text{dt}}+\frac{\text{p}-{\text{p}}_{\text{b}}}{\text{R}}=\text{I}(\text{t})+\sigma \frac{\text{dW}}{\text{dt}}$$

Rearranging the above terms yields our final model, which we will call the stochastic Marmarou model.

(9) $$\text{dp}=\{\text{Ep I}(\text{t})-\frac{\text{Ep}(\text{p}-{\text{p}}_{\text{b}})}{\text{R}}\}\text{dt}+\sigma \text{Ep dW}$$

Note that in order for equation (9) to be dimensionally consistent, the unit of σ is mL/min. Because the 'input' I(t) is the infusion rate which is under direct experimental control, therefore, in the language of control theory, I(t) is a 'control' variable. In the infusion studies conducted at Addenbrookes's Hospital in Cambridge, UK, I(t) is maintained at a constant rate of 1.5 mL/min. However, factors not within the experimenter's control also influence the input flow rate. In addition to the infusion rate of the experimenter which influences CSF formation, CSF is produced inside the brain, but much about its production remains unknown at the present time. Currently, there are no direct methods to measure the CSF production rate over short periods of time. Globally, the average secretion rate--used as a proxy for the production--is 0.35 mL/min with a 95% confidence range of 0.27 mL/min to 0.45 mL/min [2]. The lack of precise knowledge about the CSF production rate and the unmeasured factors that influence it are sources of noise in the total CSF formation rate. Consequently the stochastic Marmarou model may be conceptualized as the classic Marmarou model with a noisy input flow rate that reflects uncertainty about CSF formation.

**Figure 3:**
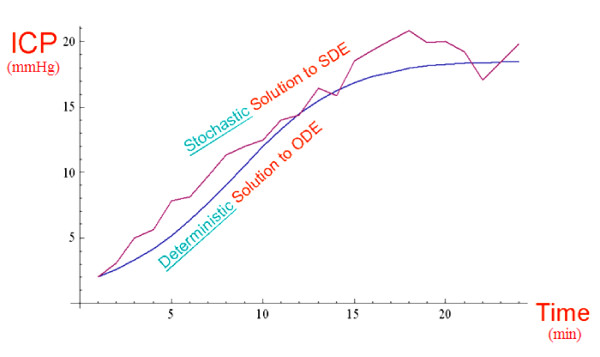
**Comparison of deterministic and stochastic Marmarou model solutions**. Values of CSF flow parameters used in this simulation: E = 0.15 mL^-1^, p_b _= 8 mmHg, R = 7 mmHg/(mL/min), I = 1.5 mLmin^-1^, σ = 0.5 mL/min.

The deterministic Marmarou model is contained in the final model displayed above--it surfaces when σ = 0 mL/min, which precludes noise, and consequently produces only the mean ICP value. The general model with σ ≠ 0 mL/min reproduces the fluctuations inherent in the time-path of real measurements of ICP--information which is discarded by the deterministic Marmarou model. Figure 3 compares the fluctuating path, similar to the actual noisy ICP data, reproduced by the stochastic Marmarou model with the path predicted by the deterministic Marmarou model. The mathematical structure of the Marmarou et al. [5] equation is the classic Verhulst logistic model, ubiquitous in biological growth and saturation phenomena [27]. The mathematical form of equation (9) is the stochastic logistic model and it is the natural stochastic extension [28,29] of the Verhulst logistic model.

### The clinical significance of the stochastic Marmarou model

By building the fluctuations right into the dynamics of the model structure, the stochastic model makes full use of the information in the variations of the ICP waveform. From this additional information, the time-varying probability distributions of the ICP waveform can be extracted, and it is these latter quantities that enable computation of the probabilities of clinically relevant events. It is the knowledge of these probabilities of clinically relevant events that facilitate dynamic risk management of the patient. Conceptually, the average value of p(t) at any given time 't' is the average ICP at that time in an ensemble of patients with a similar CSF flow profile, as reflected in the values of the CSF flow parameters.

### Analyzing the stochastic Marmarou model

In the Results section, we will display the exact analytical solution to the stochastic Marmarou model and derive insights from the solution into the influence of noise on the ICP at each point in time, and on average. Under the normal conditions described in [2], biological processes will settle down to a steady state after the transients have died out. In the deterministic Marmarou model [5], the steady state (equilibrium) is found by setting the time rate of change of the ICP equal to zero. What is the corresponding steady-state concept for a stochastic process? The stochastic counterpart to the time-independent steady-state level of the ICP is the time-independent probability distribution of the ICP, and the equilibrium probability distribution is to the stochastic environment as the stable equilibrium point is to the deterministic one [30]. We derive the equilibrium probability distribution for the ICP, and from it, draw conclusions for the influence of CSF flow parameters and noise intensity upon the average steady-state ICP level. We compute a measure relevant to the treatment and control of hydrocephalus: given the current value of the patient's ICP, what is the probability that it will exceed a critical high level? And how is that probability influenced by neurological characteristics of the patient such as their resistance to CSF flow and the noise intensity of the fluctuations in CSF formation rate which in turn drives the fluctuations in their ICP?

### Computing probabilities of clinically relevant events

The mathematical formulation of the problem posed in the previous paragraph is: given that a patient's ICP is currently x mmHg, where x is an arbitrary value, what is the probability that the ICP will exceed a critical threshold 'b' (mmHg) at a future time? Mathematically stated: given that p(s) = x, find the following transition probability-- P[p(t) > b | p(s) = x], t > s. Simple though the question seems, finding the answer requires computing the conditional probability distributions of the CSF process. Since the conditional probability distributions follow the Fokker-Planck partial differential equation, the problem is non-trivial, but Karlin and Taylor [31] circumvent the difficulty by solving a boundary-value problem associated with this dynamically changing probability. They show that the required probability satisfies a nonlinear ordinary differential equation which must be solved subject to two conditions on the probability that are natural consequences of the current ICP level when it is at one of the two extreme points of the range of ICP values under consideration. It is these conditions that give rise to the term 'boundary value problem.'

## Results and Discussion

We state and discuss the significance of the mathematical results, deferring their proofs to the Appendices (Additional file 1, Additional file 2 and Additional file 3) in the interest of maintaining clarity of exposition. Our first result is the exact analytical solution to the stochastic Marmarou model.

### Solution to stochastic Marmarou model with constant rate infusion

For a constant infusion rate I, equation (9) is explicitly solvable in closed-form as shown below. Given any initial ICP value "p_0_" (mmHg) at time t = 0, the future ICP value at any time "t" is given by:

$$\text{p}(\text{t})=\frac{\exp\left[\left\{\text{E}\left(\text{I}+\frac{{\text{p}}_{\text{b}}}{\text{R}}\right)-\frac{\sigma^{2}{\text{E}}^{2}}{2}\right\}\text{t}+\sigma \text{E W}(\text{t})\right]}{\frac{1}{{\text{p}}_{0}}+\frac{\text{E}}{\text{R}}\int_{0}^{\text{t}}\exp\left[\left\{\text{E}\left(\text{I}+\frac{{\text{p}}_{\text{b}}}{\text{R}}\right)-\frac{\sigma^{2}{\text{E}}^{2}}{2}\right\}\text{s}+\sigma \text{E W}(\text{s})\right]\text{ds}}$$

The proof is provided in additional file 1: Solving the stochastic Marmarou model. Note that the solution to the stochastic Marmarou model is found through an "integrating factor" which involves an integration constant, the evaluation of which necessitates a unit of 1/min unit for the 2 inside the exponent of the exponential function. The noise intensity parameter σ and the Brownian Motion process W(t) in the solution show the explicit influence of noise on the evolution of the ICP, underscoring the importance of modeling the noise in the clinical ICP data. In addition to the practical utility of offering a closed-form analytical solution, this result has value for another reason: it shows explicitly that noise cannot be averaged away when the process is nonlinear. If the Brownian motion process W(t) entered the solution for p(t) in an additive linear way, its effect would disappear on average. But the Brownian motion process enters the solution in a highly nonlinear fashion, making it impossible to average out its effect to zero. Finally, the solution depends upon the noise intensity parameter σ in a mathematically continuous way, a fact that is meaningful because the result shows that the solution to the deterministic Marmarou model [5] emerges as the special case corresponding to σ = 0 mL/min, and so, it is natural to ask if the simpler deterministic model would suffice when the noise intensity is small. Should the influence of noise be negligible in a particular case, the value of σ will be very small, and, because of the mathematical continuity in its dependence upon σ, the stochastic solution will be very close to the deterministic solution in such a case, and we may use the simpler deterministic model with confidence. However, the stochastic model is preferable in general for two reasons: it captures the dynamics of the ICP data better than the deterministic model when the noise intensity is larger, and furthermore, the stochastic model characterizes the risk profile of the patient probabilistically. Almost tautologically, the deterministic model cannot evaluate the risks due to the errors that are an inseparable part of medical data because deterministic modeling philosophy sees the future as completely predictable from the present situation. These considerations suggest that, from a conservative modeling perspective, incorporating the influence of noise into the dynamics is conceptually more defensible.

In principle, the solution contains all the transient probability distributions of the ICP process that characterize it on its way to equilibrium. In practice, mathematical difficulties may make these transient distributions hard to extract from the solution. But we can still compute the probability of the critical events by using a methodology that does not depend on that knowledge. And we can still draw useful information about the nature of the process at steady-state. Next, we find the steady-state probability distribution of the ICP process.

### Steady-state probability distribution of ICP

The steady-state probability distribution of the ICP is gamma with the parameters shown p.149 in [29], and will exist provided that the noise intensity parameter σ satisfies the condition: $\sigma^{2}<\frac{2\left(\text{RI}+{\text{p}}_{\text{b}}\right)}{\text{RE}}$.

**Figure 4:**
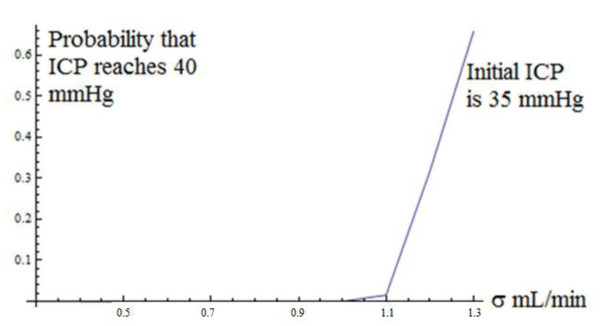
**Probability that ICP reaches 40 mmHg as a function of noise intensity parameter σ, starting at an ICP level of 35 mmHg**. Values of CSF flow parameters used in this simulation: E = 0.15 mL^-1^, p_b _= 8 mmHg, R = 7 mmHg/(mL/min), I = 1.5 mLmin^-1^, σ ranged from 0.4 mL/min to 1.3 mL/min.

The proof is provided in additional file 2: Finding the steady-state probability distribution of the ICP. The transition probability function satisfies the Fokker-Planck partial differential equation, which at steady-state, becomes an ordinary differential equation (ODE). The solution to the Fokker-Planck ODE yields the steady-state probability density function, but an arbitrary constant appears in the solution whose value must be such that the total area under the steady-state probability density function is unity. The integration required to evaluate the normalization constant generates a unit of 1/min for the 2 in the above inequality. The mean and variance of the gamma distribution are not independent parameters as they are for the normal distribution. Unlike the normal distribution in which the mean is the location parameter and the variance is the shape parameter, neither of the parameters of the gamma distribution is a pure location or pure shape parameter. Thus, the two parameters that characterize the gamma distribution *jointly *determine its location and shape, because the mean and variance of this distribution are functions of *both *the parameters. In practice, biological phenomena will converge to a steady-state, but nonetheless it is important to check that the technical condition stated for the existence of a steady-state distribution is satisfied by realistic values of the Marmarou model parameters. We obtained typical values of R, E, p_0_, p_b _and I from a combination of [2] and private communication with Dr. M. Czosnyka, yielding R = 7 mmHgmL^-1 ^min (reported values range between 6 and 10 mmHgmL^-1 ^min), p_0 _= 0 mmHg and p_b _= 8 mmHg. Elevated elasticity is reported to be E > 0.18 ml^-1 ^and the rate of infusion is I = 1.5 mLmin^-1 ^[2]. The value of E was taken to be E = 0.15 mL^-1^, based on private communication with Dr. Czosnyka, and this value came from data gathered in infusion studies conducted at Addenbrooke's Hospital. p_b _is a baseline pressure which is different for each individual patient. Based on the ICP plots in [2], we set p_b _= 8 mmHg. This value is close to the average p_b _across all infusion studies conducted at Addenbrooke's Hospital which Dr. Czosnyka, in private communication, reported to be 6 mmHg. While the authors solved the deterministic Marmarou model for the general case of p_0 _≠ 0 mmHg in [2], and found that the average value of p_0 _in the infusion studies was p_0 _= 4 (private communication with Dr. Czosnyka), the non-zero p_0 _case is currently not analytically solvable for the stochastic Marmarou model. Our p_0 _= 0 mmHg assumption is consistent with [5], in which the authors ignore the reference pressure. However, we acknowledge that the non-zero p_0 _case is an important issue in mathematical modeling of hydrocephalus--and the author and his collaborators are working on an algorithm to solve the stochastic Marmarou model for non-zero p_0_. A typical value for σ is difficult to find since the input flow rate of CSF is not accessible to direct observation--only the fluctuations in the ICP are observable. We estimated σ roughly as, σ = 0.33*I = 0.33*1.5 mL/min = 0.5 mL/min, so that the flow fluctuations are 33% (1/3) of the flow rate. This is a rough estimate--the choice of a typical value for σ is unclear. Because of the uncertainty and approximations involved in the estimate, we did the computations in which σ was fixed, not just at σ = 0.5 mL/min, but also at values of σ lower as well as higher than 0.5 mL/min in order to check the robustness of the conclusions. We have reported the results for σ = 0.5 mL/min and for σ = 0.8 mL/min. The results of the computations of the probability as a function of R are robust across a wide range of σ, so that, even though the estimate of σ is only approximate, we can be reasonably confident about the conclusions of the analysis. To examine the influence of σ itself on the risk probability, we computed the probability across a wide range of σ as shown in Figure 4--again, in an effort to reduce the impact of our imprecise knowledge of σ upon the findings.. While our estimate of σ is only approximate, we note that there is imprecision and uncertainty about *all *the parameter estimates, especially that of the CSF flow resistance 'R.' Estimation methods specifically developed for dynamic models are needed. In this paper, the primary objectives were to introduce SDE methodology to CSF research, demonstrate its analytical power, and show its clinical usefulness in dynamic risk management. Consequently, we used existing typical estimates of the model parameters even though some of them are imprecise and approximate. With σ = 0.5 mL/min, the condition for the existence of a steady-state probability distribution is met with ease.

Our next three results are motivated by the following considerations. A larger cerebrospinal fluid resistance R tends to increase ICP by increasing the pressure due to the circulatory CSF component. This is a direct consequence of Davson's equation [6]: ICP_CSF _= (resistance to CSF outflow) × (CSF formation) + (pressure in sagittal sinus). This naturally leads to the following questions. How will the intensity of the fluctuations influence the relationship between resistance and ICP? The same relationship may hold on average, but, as anticipated in the solution to the stochastic Marmarou model, it may be moderated by the noise intensity parameter because of the nonlinearity of the ICP process. How will the intensity of fluctuations affect the average steady-state ICP--is the average steady-state ICP smaller or larger when the intensity of fluctuations increases? Finally, will the intensity of fluctuations attenuate or amplify the effect of resistance to CSF flow on the average steady-state ICP?

### Relationship between average steady-state ICP and cerebrospinal fluid resistance

The average steady-state ICP, denoted by μ, increases with the cerebrospinal fluid resistance R--thus the relationship between R and ICP holds on average.

The steady-state probability distribution of ICP is gamma with the parameters shown in the previous subsection. From well-known properties of the gamma distribution, it follows that the steady-state mean ICP level μ is given by: $\mu =\left(\text{RI}+{\text{p}}_{\text{b}}\right)-\frac{\text{RE}\sigma^{2}}{2}$. Therefore, $\frac{\partial \mu }{\partial \text{R}}=\text{I}-\frac{\sigma^{2}\text{E}}{2}$. From the expression for $\frac{\partial \mu }{\partial \text{R}}$, it is clear that the average ICP level does indeed increase with R, provided that $\frac{\sigma^{2}\text{E}}{2}<\text{I}$. This condition is satisfied, using the values of the parameters in the previous subsection. Thus, the increasing relationship between the actual ICP level and the cerebrospinal fluid resistance, predicted by Davson's equation when ICP is conceptualized as a deterministic process, also holds *on average *at steady-state when ICP is modeled as a stochastic process.

### Relationship between average steady-state ICP and noise intensity

The average steady-state ICP level, decreases with the intensity of fluctuations, measured by the infinitesimal variance parameter σ^2^.

From the relationship derived in the previous subsection, $\mu =\left(\text{RI}+{\text{p}}_{\text{b}}\right)-\frac{\text{R}\sigma^{2}\text{E}}{2}$, it is clear that μ decreases as σ^2 ^increases. A larger noise intensity corresponds to greater variation in the CSF input flow rate which translates into greater variation in ICP, and these larger fluctuations could cause the average ICP level to increase, decrease or remain unaffected. The nonlinear influence of the parameters of CSF flow dynamics on ICP level turns out to reduce the average ICP value when the fluctuations in ICP are greater. This is an outcome that one would expect to find when steady-state has been achieved--when the transition probabilities have settled down to constant levels so that the probability distribution of ICP is no longer changing over time. This mathematical finding could be tested by separating a random sample of patients into two groups, such that one group has more variability in its ICP levels (due to higher variability in its CSF input flow rate) than the other group, and then conducting a statistical test of significance--such as a t-test--on the difference in mean ICP levels in these two groups at steady-state.

### Effect of noise intensity on the relationship between average steady-state ICP and cerebrospinal fluid resistance

The resistance increases the ICP on average by a smaller amount when the intensity of fluctuations is higher.

From $\mu =\left(\text{RI}+{\text{p}}_{\text{b}}\right)-\frac{\text{R}\sigma^{2}\text{E}}{2}$, it is clear that a higher σ^2 ^will dampen the effect of the cerebrospinal resistance on the average steady-state ICP level. This is an outcome that one would expect to find at steady-state. The mathematical finding could be tested by separating a random sample of patients into two groups, such that one group has more variability in its ICP levels than the other group (due to higher variability in its CSF input flow rate), and then correlating the mean ICP level with the cerebrospinal resistance in each group at steady-state. According to the mathematics, the correlation should be smaller in the group with more variable ICP. Given the linear relationship between the steady-state mean and the cerebrospinal resistance, a simple correlation coefficient such as the Pearson product moment should suffice.

Next we turn our attention to dynamic management of the patient's risk. Risk may be quantified in terms of the probability of the onset of some critical event, say the ICP exceeding a dangerously high level. Given the current value of the patient's ICP, what is the probability that it will exceed a high level? Such a probability is intrinsically dynamic because it depends upon the patient's current condition (their current ICP), the dynamics of the patient's CSF flow and the noise intensity σ^2^. We want to understand how the probability is influenced by important clinical characteristics of the patient such as their resistance to CSF flow, and by the noise intensity.

### Computing clinically relevant dynamic probabilities

Given that the current ICP is x mmHg, where 0 ≤ × ≤ b, let u(x) denote the probability of reaching the level b. Then u(x) satisfies the following nonlinear differential equation, which must be solved subject to the two conditions on u(x) at x = 0 and at x = b:

$$\begin{array}{l} \frac{\text{du}}{\text{dx}}\{\text{EIx}-\frac{\text{Ex}(\text{x}-{\text{p}}_{\text{b}})}{\text{R}}\}+\frac{{\text{d}}^{2}\text{u}}{{\text{dx}}^{2}}\frac{\sigma^{2}{\text{E}}^{2}{\text{x}}^{2}}{2}=0\\ \text{u}(0)=0,\text{ u}(\text{b})=1 \end{array}$$

The conditions on u(x) at the two corners x = 0 and x = b make this a two-point boundary value problem. The solution is given in terms of the scaling function S(x):

$$\text{u}(\text{x})=\frac{\text{S}(\text{x})-\text{S}(0)}{\text{S}(\text{b})-\text{S}(0)}\text{, where S}(\text{x})=\int_{}^{\text{x}}\text{s}(\eta )\text{d}\eta \text{, and s}(\text{x})=\exp\left[-\int_{}^{\text{x}}\frac{2\left[\text{EI}\xi -\frac{\text{E}\xi (\xi -{\text{p}}_{\text{b}})}{\text{R}}\right]}{\sigma^{2}{\text{E}}^{2}\xi^{2}}\text{d}\xi \right].$$

The integrals defining s(x) and S(x) are indefinite at the lower end because the final answer is unaffected by its choice. For our clinical applications, it is natural to take the lower end point to be zero.

**Figure 5:**
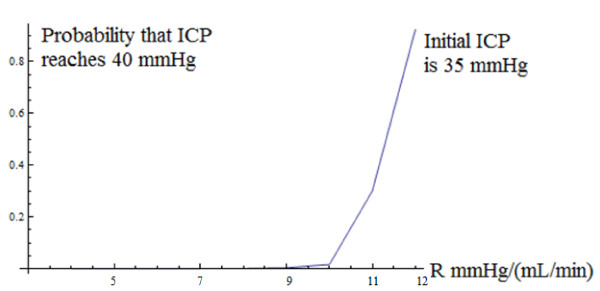
**Probability that ICP reaches 40 mmHg as a function of resistance to CSF flow parameter R, starting at an ICP level of 35 mmHg**. Values of CSF flow parameters used in this simulation: E = 0.15 mL^-1^, p_b _= 8 mmHg, I = 1.5 mLmin^-1^, σ = 0.8 mL/min, R = ranged from 4 mmHg/(mL/min) to 12 mmHg/(mL/min).

**Figure 6:**
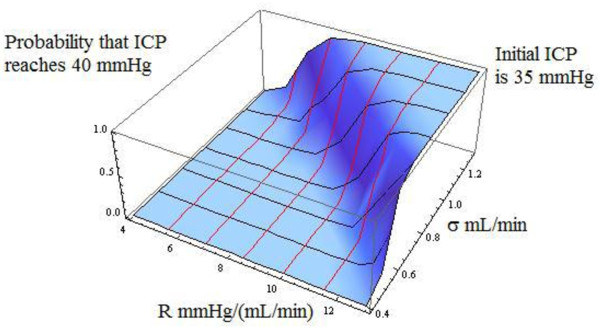
**Probability that ICP reaches 40 mmHg as a function of resistance to CSF flow parameter R, and noise intensity parameter σ, starting at an ICP level of 35 mmHg**. Values of CSF flow parameters used in this simulation: E = 0.15 mL^-1^, p_b _= 8 mmHg, I = 1.5 mLmin^-1^, R = ranged from 4 mmHg/(mL/min) to 12 mmHg/(mL/min), and σ ranged from 0.4 mL/min to 1.3 mL/min.

The proof is deferred to additional file 3: Computing clinically relevant dynamic probabilities. While the above representation is, in principle, a closed-form analytical solution, it is, in practice, a *quasi-analytical *solution because the integral that defines s(x) cannot be obtained in closed-form. However, that is no limitation because we can integrate it numerically after substituting the empirically established values of the parameters. We used the parameter values shown in the subsection "Steady-state Probability Distribution of ICP." We took the critical level 'b' to be 40 mmHg, based on the clinical finding reported in [2] that patients were able to tolerate increases in ICP up to 40-50 mmHg. Our rationale was that, from a clinical perspective, a conservative approach to patient management would be consistent with assessing the probability of reaching the lower end point of the 40-50 mmHg range that patients are able to tolerate. Thus our critical event is defined as "reaching an ICP of 40 mmHg." In order to understand how the probability is influenced by the noise intensity parameter σ, we computed the probability over a range of σ = 0.4 mL/min to σ = 1.3 mL/min. For each value of σ in this range, we solved the boundary-value problem to find the probability of reaching 40 mmHg. Furthermore, in order to understand the influence of the patient's initial condition upon the probability of the critical event, we repeated this set of computations for three different starting levels of ICP; the curve shown in Figure 4 is for a starting level of ICP of 35 mmHg. In order to understand how the probability is influenced by the resistance to CSF outflow R, we computed the probability over a range of R = 4 mmHgmL^-1 ^min to R = 12 mmHgmL^-1 ^min. For each value of R in this range, we solved the boundary-value problem to find the probability of reaching 40 mmHg. Again, we repeated this set of computations for three different starting levels of ICP; the curve shown in Figure 5 is for a starting level of ICP of 35 mmHg. Across the three initial levels of ICP, the curves have a similar shape and are simply translated vertically. Figures 4 and 5 show that the probabilities increase at an increasing rate (convex functions). Furthermore, they tell an interesting neurological story--namely, that the probabilities of the critical events exhibit strong *threshold *effects. In Figure 4, below a critical level of noise intensity, the probabilities are very low--almost zero--but beyond a threshold value of σ = 1.1 mL/min in Figure 4, they rise steeply. In Figure 5, as R (mmHgmL^-1^min) varies from 4 to below 10, the probabilities are almost zero, but beyond R = 10 mmHgmL^-1 ^min, they rise dramatically. Furthermore, at low levels of noise intensity, the probabilities remain close to zero *throughout *the range of R (mmHgmL^-1^min) from 4 to 12. But as σ increases to 0.8 mL/min--the value assumed for it in Figure 5--R has a strong effect on the probability beyond the critical threshold of 10 mmHgmL^-1^min. The clinical significance of these findings is that erratic fluctuations in ICP (caused by a larger input flow rate noise intensity σ) will significantly increase the patient's risk, as measured by the probability of the critical event. Because the risk increases rapidly beyond the threshold value of σ, these results suggest that an essential component of risk management is to carefully minimize erratic fluctuations in the patient's CSF input flow rate at all times. Finally, Figure 6 shows the probability of the critical event as a function of both R and σ in a three-dimensional plot. The two-dimensional surface shows the value of the probability for each combination of values of R and σ. To facilitate interpretation of the surface, we used a mesh in which the dark lines are the probability plots as a function of R and the red lines are the probability plots as a function of σ. Figure 6 shows very clearly that threshold effects are sensitive to both R and σ, and beyond the threshold, the probabilities asymptotically approach one.

## Conclusions

The stochastic generalization of the Marmarou model offers a tractable analytical description of the noisy ICP dynamics and yields insights into the impact of noise. The SDE offers a rigorous analytical framework to study issues of clinical interest and neurological significance such as the patient's risk. A key clinical implication is that fluctuations in the CSF formation rate--which increase the fluctuations in ICP-- should be minimized to lower the patient's risk. Future work could extend the framework developed in this research to accommodate the non-zero reference pressure case. Finally, the stochastic differential equation framework, in conjunction with nonlinear control theory, can be used to develop a nonlinear automatic controller to regulate shunts to facilitate continuous CSF drainage.

## Competing interests

The author declares that they have no competing interests.

## Authors' contributions

KR is the sole author. He has read and approved the final version of the manuscript.

## Supplementary Material

Additional file 1 **Solving the stochastic Marmarou model**. This file derives the solution to the stochastic Marmarou Model assuming a constant infusion rate. Click here for file

Additional file 2 **Finding the steady-state probability distribution of the ICP**. This file derives the steady-state probability distribution of the ICP. Click here for file

Additional file 3 **Computing clinically relevant dynamic probabilities**. This file shows how to derive the dynamic probability of the critical event--defined as the ICP exceeding the critical threshold level of ICP of 40 mmHg, as a function of the patient's current ICP level, the baseline pressure, the patient's neurological characteristics-- the resistance to CSF flow, the cerebral elasticity, and an experimental variable--the infusion rate. Click here for file
