# Supplementary material for: A stochastic differential equation analysis of cerebrospinal fluid dynamics
Source: Fluids Barriers CNS. 2011 Jan 18;8:9. doi: 10.1186/2045-8118-8-9 (PMC3042983; doi:10.1186/2045-8118-8-9)
Supplement: Additional file 1 — Solving the stochastic Marmarou model. This file derives the solution to the stochastic Marmarou Model assuming a constant infusion rate. [file 2045-8118-8-9-S1.PDF]

Appendix 1

Proof of Solution to stochastic Marmarou Model with Constant Infusion: The stochastic Marmarou model with a constant infusion rate is

$$dp = \{EIp - \frac{Ep(p - p_b)}{R}\}dt + \sigma Ep\,dW$$

This may be rewritten in the form of the stochastic logistic model

$$dp = \left(\frac{E}{R}\right)p\left(RI + p_b - p\right)dt + \sigma Ep\,dW$$

It is shown in [28, p.78] that the solution to the stochastic logistic model

$$dX = rX(K - X)dt + \sigma X\,dW$$

is

$$X(t) = \frac{\exp\left[\left\{rK - \frac{\sigma^2}{2}\right\}t + \sigma W(t)\right]}{\frac{1}{x_0} + r\int_0^t \exp\left[\left\{rK - \frac{\sigma^2}{2}\right\}s + \sigma W(s)\right]ds}$$

Identification of the parameters "r" with (E/R), 'K' with ($RI + p_b$) and σ with σE produces the claimed solution.
