# Supplementary material for: A stochastic differential equation analysis of cerebrospinal fluid dynamics
Source: Fluids Barriers CNS. 2011 Jan 18;8:9. doi: 10.1186/2045-8118-8-9 (PMC3042983; doi:10.1186/2045-8118-8-9)
Supplement: Additional file 2 — Finding the steady-state probability distribution of the ICP. This file derives the steady-state probability distribution of the ICP. [file 2045-8118-8-9-S2.PDF]

Appendix 2

Proof of Steady-state Probability Distribution of ICP:

Let $\phi(x,t,y,s) = P[p(t) \in (x, x+dx) \mid p(s) = y]$ be the transition density.

Then, $\phi(x, t, y, s)$ satisfies the Fokker-Planck partial differential equation, hereafter abbreviated (FPE), stated in [29, p.30] for a general vector stochastic process, and here specialized to a scalar process for the stochastic Marmarou model:

$$\frac{\partial\phi}{\partial t} = -\frac{\partial[f(p)\phi]}{\partial p} + \frac{1}{2}\frac{\partial^2[g(p)^2\phi]}{\partial p^2}$$

subject to

$\phi(x, t, y, s) = \delta(x - y)$, where $\delta(x - y)$ is the generalized Dirac-delta function centered at y. The FPE shows that the transition probabilities vary over time during the transient phase, but at steady-state, the probability transition functions are time-independent, and consequently $\frac{\partial\phi}{\partial t} = 0$.

The Fokker-Planck partial differential equation then becomes an ordinary differential equation which may be solved to find the steady-state distribution of the ICP process p(t). The ODE is shown below:

$$\frac{d}{dp}\left[-f(p)\phi + \frac{1}{2}\frac{d[g(p)^2\phi]}{dp}\right] = 0$$

Solving the time-independent Fokker-Planck ODE yields the Gamma distribution with parameters that are shown in [29, p. 149]. It is shown in [29, p.149] that the steady-state distribution for the SDE $dX = aX(1-\frac{X}{K})dt + bXdW$ will exist provided that $b^2 < 2a$. The

condition for the steady-state distribution of the ICP stated in the paper follows upon appropriate identification of the parameters of the stochastic Marmarou model with those of the model in [29].
