# Supplementary material for: A stochastic differential equation analysis of cerebrospinal fluid dynamics
Source: Fluids Barriers CNS. 2011 Jan 18;8:9. doi: 10.1186/2045-8118-8-9 (PMC3042983; doi:10.1186/2045-8118-8-9)
Supplement: Additional file 3 — Computing clinically relevant dynamic probabilities. This file shows how to derive the dynamic probability of the critical event--defined as the ICP exceeding the critical threshold level of ICP of 40 mmHg, as a function of the patient's current ICP level, the baseline pressure, the patient's neurological characteristics-- the resistance to CSF flow, the cerebral elasticity, and an experimental variable--the infusion rate. [file 2045-8118-8-9-S3.PDF]

Appendix 3

Proof of Solution to Computing Clinically Relevant Dynamic Probabilities: Consider the stochastic differential equation $dX = \mu(X)\,dt + \sigma(X)\,dW$. Given that the current value of X(t) is x, where $0 \le x \le b$, let u(x) denote the probability of reaching the level b. It is shown in [31, p.193] that u(x) satisfies the nonlinear ordinary differential equation:

$$\frac{du}{dx}\mu(x) + \frac{d^2u}{dx^2}\frac{\sigma^2(x)}{2} = 0$$
$$u(0)=0,\ u(b)=1$$

Therefore, in the context of this paper, u(x) satisfies the following ODE,

$$\frac{du}{dx}\{EIx - \frac{Ex(x-p_b)}{R}\} + \frac{d^2u}{dx^2}\frac{\sigma^2E^2x^2}{2} = 0$$
$$u(0)=0,\ u(b)=1$$

Using Green's function methods, the solution to the above boundary value problem is given in terms of an important quantity called the scaling function S(x) [31, p.194-195]:

$$u(x) = \frac{S(x)-S(0)}{S(b)-S(0)}, \text{ where } S(x) = \int^{x} s(\eta)\,d\eta, \text{ and } s(x) = \exp\left[-\int^{x}\frac{2\mu(\xi)}{\sigma^2(\xi)}d\xi\right].$$

Identification of the parameters with those of the stochastic Marmarou model,

$$\mu(p) = \left(\frac{E}{R}\right)p\left(RI + p_b - p\right),\ \sigma^2(p) = \left(\sigma Ep\right)^2$$

immediately yields the claimed result in this subsection of the paper.
